# Detecting informal green, blue, and street physical activity spaces in the city using geotagged sports-related Twitter tweets

**DOI:** 10.3389/fsoc.2023.1125343

**Published:** 2023-05-05

**Authors:** Charlotte van der Lijn, Emil Ehnström, Sonja Koivisto, Petteri Muukkonen

**Affiliations:** ^1^Department of Geosciences and Geography, University of Helsinki, Helsinki, Finland; ^2^School of Geography and Sustainable Development, University of St. Andrews, St. Andrews, United Kingdom

**Keywords:** geotag, informal sports, physical activity, Twitter, urban planning, wellbeing

## Abstract

**Introduction:**

Finland's natural physical environment and climate support a wide variety of informal outdoor sports, thereby motivating the population to do physical exercise in scenic environments. The vast majority of Finns enjoys outdoor recreational activities, and could thus be encouraged to post accounts of their year-round activities on social media. Our aim was to find out in what kind of areas and spaces, spatially, users are tweeting about sporting activities.

**Methods:**

We use geotagged Twitter tweets filtering for 16 sporting activity keywords in both English and Finnish. The case study was conducted in the Helsinki Metropolitan Area, Finland, with an emphasis on cross-country skiing as a sports activity when there is snow. In a secondary analysis we concentrated on the sports people were practicing in these locations when there was no snow. The location spaces are split in to three land cover types: green, blue, and street spaces.

**Results:**

We found that approximately half of the 150 skiing-related tweets were geotagged in green spaces, and half in street spaces. This finding related to street space was attributable to a spatial scale error: when we checked the results manually we noticed that they referenced the sporting location in the green space. Hence, then over 90% of the 745 non-ski-related tweets were geotagged in a street space.

**Discussion:**

We conclude that Twitter is a beneficial tool for detecting spaces used for informal physical activity. A shortcoming in current Finnish national sporting policies is that spaces for informal physical activity are not explicitly mentioned- we use the term informal with reference both to the space and to the sporting activity, whereby public spaces are used for physical activity. This new knowledge of sporting locations will help city planners and sports planners to improve informal sports facilities, which in turn will promote healthy exercise in cities.

## 1. Introduction

There is an abundance of free informal outdoor space for physical activity (PA) in Finland, ranging from volleyball on the beach to ice skating on the frozen sea and lakes. We interpret informal sport to encompass any urban and nature-based lifestyle PA that takes place in a publicly accessible space away from fee-paying official sports facilities. Although informal sport is not a new concept in academic literature, as Chalip et al. (1984) already having studied the overall enjoyment of participation, it was only quite recently that it began to gain traction in official policy making, such as reported in Jeanes et al. (2019). This strengthening of focus in policy making could reflect the fact that participation in informal sport is said to be in decline (Coakley, 2017).

Therefore, as we point out in this article, there is a need to investigate where people engage in PA to establish the demand in urban locations. We use social-media data, coming from public geotagged Twitter messages, meaning that the location attribute is already attached to the messages. Our main analysis focuses on key words related to cross-country skiing, and is geographically limited to the Helsinki Metropolitan Area (HMA) in southern Finland. The secondary analysis concerns other sporting activity in the same locations as in the skiing tweets. The HMA comprises the cities of Helsinki, Vantaa, and Espoo, and the town of Kauniainen. These four areas combined constitute the largest urban concentration in Finland. It is not a typical urban area in that it contains many urban green (such as public parks, forests, or nature reserves) spaces and urban blue (such as rivers, lakes, or the sea) spaces that are suitable for PA. Additionally, the majority of Finnish cities are designed to allow everyone access to places where they can engage in PA, regardless of whether or not they like to do so outdoors (Neuvonen et al., 2018).

Our main research question is: “How can geotagged Twitter tweets detect informal PA spaces?” and our sub-question is “In what type of urban public space do people engage with in their informal PA?.” We split the tweet analysis by skiing related key words in both English and Finnish, whereby skiing in Finland means cross-country skiing but can also include downhill skiing if done at a physical facility. Skiing in the HMA is an interesting research topic because, even though it is an urban environment, there are numerous opportunities for skiing in public green, blue, and even street PA spaces. We extend the analysis by comparing the skiing tweets to other sporting tweets within the same location to find out what sports people engage in when there is no snow.

The literature on informal participation in sport focuses on the improvement of overall health and wellbeing (Bowler et al., 2010). We do not separate the terms “sport,” “exercise,” and “participation” here, because in our view any type of PA is beneficial in terms of having an active and healthy lifestyle in the city. We therefore use the term “physical activity” from here on, following the World Health Organization (WHO) definition: “physical activity refers to all movement including during leisure time, for transport to get to and from places, or as part of a person's work” (WHO, 2020). Children in Finland are encouraged to engage in outdoor play from a young age, going outside in all weathers while at kindergarten (Iivonen et al., 2019). Furthermore, our case study area, the HMA, has many outdoor play parks, averaging almost one per neighborhood, which is not the norm in many other countries. These parks promote exercise in natural environments whilst honing children's independent mobility through to adulthood (King and Sills-Jones, 2018). However, even though Finns seem to have an ingrained habit of engaging in PA year-round, around 70% of the adult population (over 18 years old) are not reaching the recommended weekly target of ≥150 min activity of moderate-to-vigorous-intensity PA (Bennie et al., 2017).

Kolu et al. (2022) point out the economic burden of a sedentary lifestyle in Finland: the total cost of low PA behavior came to approximately € 3.2 billion in 2017, and the costs attributable to high sedentary behavior totaled around € 1.5 billion. According to the Finnish Institute for Health and Welfare (2022), around 70% of men and 60% of women in Finland are overweight, meaning that they have a body mass index (BMI) of 25 or more, and a third of the population are obese, having a BMI of 30 or more: a score of 18.5–24.9 is healthy. It was found in a similar recent study conducted by the Helsinki and Uusimaa Hospital District that the annual healthcare costs for an obese person were approximately € 2,665 (Vesikansa et al., 2022). Rose et al. (2008), in turn, reported that spending two or more hours outdoors helps to prevent children from becoming short sighted in the near future, which also eases the burden on health care. Therefore, the provision of free public spaces that allow the population to be active outdoors is one among many simple and effective ways of improving health and simultaneously lowering the negative costs attached to being inactive. It is suggested in current literature based on PA rates and the impact of the COVID-19 pandemic that there is a general tendency to be less active (e.g., Engels et al., 2021; Ronkainen et al., 2021; Semple et al., 2021). However, according to the Ministry of the Environment (2021) there is a strategy to increase the recreational use of natural habitats in Finland as part of Prime Minister Sanna Marin's government program. Survey results have shown that, in fact, the COVID-19 pandemic increased the motivation of Finns to exercise in local nature-based surroundings, whereby almost everyone (96%) enjoys outdoor activities: this adds to the relevance of our article.

Researchers have found it difficult to establish participation rates for informal lifestyle sport (Gilchrist and Wheaton, 2011). PA of this type does not typically go on in a fixed place such as a physical sports facility, hence usual practices such as counting tickets are not possible. Consequently, for this article we focused our analysis on already geolocated Twitter tweets to detect the demand for informal sport by location, meaning that the PA takes place in an informal setting away from physical sports facilities. It should be borne in mind that not all PA takes place by or in green or blue urban spaces, and that street spaces also exist. Finland has a national database of sports facilities (LIPAS, 2022), which also includes informal sports infrastructure comprising street spaces (any location not in green or blue spaces) such as outdoor table-tennis tables and basketball courts. We therefore distinguish between three informal urban PA spaces which are also land cover types: (1) green, (2) blue, and (3) street.

## 2. Background

### 2.1. Defining informal spaces for physical activity

For the purpose of this article we focus on informal PA spaces, given that Finland already has a national open database of sports facilities and their conditions, namely LIPAS (LIPAS, 2022). User demand for formal PA facilities can be tracked by means of ticket purchases or by scanning sports cards, but this is not the case for informal spaces, hence it is more difficult to estimate user demand in each location. Table 1 shows varying definitions of informal sports, and whether they mention the location of the PA. We use the term “sport” rather than “physical activity” because the majority of literature refers to informal sport, the assumption being that participation is informal (Wheaton and O'Loughlin, 2017).

**Table 1:** Definitions of informal sports.

**References**	**Informal sport definition**	**Mentions location (Yes/No)**
Coakley (2017)	Informal games exist when young people come together and agree to organize themselves for the sake of having fun and maximizing action.	N
Kokolakakis et al. (2017)	The distinction between formal and informal definition is based on the frequency and context of participation- formal sport implies doing sport in a club, or through organized competition.	N
Wheaton and O'Loughlin (2017)	A defining feature of lifestyle sports is their self-organized and spontaneous nature, with participation in predominantly informal settings, often without external regulation or institutionalization.	**Y**
Deelen et al. (2018)	Typical informal and flexible sports settings are commercial health centers and gyms, with informal groups and individual participation in the public space, all of which make participants less dependent on formal structures such as membership obligations, opening hours and the availability of specific sports facilities.	**Y**
Jeanes et al. (2019)	Traditional recognized sporting forms, played by groups who are not affiliated to sporting bodies or pay membership fees.	N
Mathisen et al. (2019)	Informal sports do not require adolescents to be part of organized official sports clubs but may involve high levels of leisure time vigorous PA.	N

However, as Deelen et al. (2018) point out, participation in sport may be within sport clubs, gyms, or public spaces, with a focus on the “settings” in which the sporting activity is located. The authors also suggest that most informal sport goes on in public spaces and natural environments, and we follow this same distinction. In our case this even applies to downhill skiing slopes, which have specific opening times and require entrance tickets but are still classed as informal because the public can choose what time to visit within the specified period. Therefore, even though there is no fixed definition of informal sport, it typically simply means being self-organized. According to Coakley (2017), for example, formal sport is “rule-centered” and informal sport is “action-centered,” whereby the latter relies on group decision-making among the participants to be self-sustaining: however, this definition omits individual sports. One variation is that the difference is simply a matter of the frequency and context of participation, whereby formal sport takes place at regular times, requires club membership and involves taking part in organized games and competitions (Kokolakakis et al., 2017).

On the grounds that the distinction between formal and informal sport is not yet fully adopted in either government or non-government agencies, Jeanes et al. (2019) define formal sports as any activities that take place in affiliated sports clubs and within official sports structures, for which participants pay a fee and compete in leagues. They further define informal sports as any traditional sporting activity, such as cricket, football, or basketball, whereby participants do not pay membership fees and play in places such as local parks or community spaces. They omit sports such as surfing, skateboarding, and parkour, which they refer to as a “lifestyle” or “leisure” sport that evolved from resistance to traditional sport. According to Mathisen et al. (2019), club sport increased in Finland from 1992 to 2010, and general PA participation expanded with the introduction of new informal sports such as cross fit, hiking, and skateboarding. We focus here on the location of the informal sporting activity, which seems to have been omitted in existing definitions.

### 2.2. Interpreting green, blue, and street urban spaces

Green and blue urban spaces have been gaining traction in academic literature (see Korpilo et al., 2021; Tan et al., 2021). It is becoming increasingly common to study how and where people exercise in a city. Locations in which physical activities take place that are scenic and in natural surroundings tend to motivate people to improve their overall wellbeing (Loureiro and Veloso, 2014; Bell et al., 2015). Finland is a suitable country in which to conduct research on where people engage in PA given the large amount of land used as green and blue spaces. Green areas, specifically forests, comprise more than 75% of the country's land (Ministry of Agriculture and Forestry, 2022). Blue spaces, namely water bodies, are particularly high in recreational value among Finnish citizens. Inland water bodies such as lakes and rivers cover ~10% of the country's total surface area, accounting for 34,539 km^2^ (Ministry of Agriculture and Forestry, 2020). These two statistics exemplify the suitability of Finland for studying where people exercise outdoors in nature, as even urban spaces are encompassed by both green and blue spaces. According to a survey of around 1,250 residents of Helsinki and Tampere conducted by the Finnish Forest Research Institute, over 80% of them thought that the green spaces improved their residential area, and over 90% said their favorite place was in nature, in a forest, on a shore or in another type of green space (Tyrväinen et al., 2007). This has led to the term “green exercise,” which has recently arisen to emphasize the positive effects of combining PA and exposure to nature (Shanahan et al., 2016; Mnich et al., 2019). It also has an effect on people's emotions, particularly when walking or otherwise exercising in a forest environment (de Brito et al., 2020).

Having included three different types of informal urban space in this article, namely green, blue, and street, we thought it important to understand how others used these terms and what in particular made the PA informal compared with traditional or formal sports. We now turn to our third and final urban space, namely street space. Street-based sports have, in the recent past, been associated with rebellion against more traditional informal sport, encompassing skateboarding or parkour (see e.g., Angel, 2016; Rannikko et al., 2016). We define street space PA here as any physical activity that does not take place on or around green or blue space, but on asphalt, concrete, or similar hard surfaces, including those on which outdoor open sports parks are built. Outdoor public spaces in Finland have varying uses depending on the season. Gravel parks, for example, are used in summer for boules and football, and in winter for ice skating. There are 943 street workout parks in Finland, of which 326 (35%) are within the HMA (LIPAS, 2022), and there are many more outdoor street PA opportunities ranging from obstacle courses to frisbee golf and workout step hills (Kivistö, 2022). Indoor public sport facilities were forced to close to the public during the winter of 2020–2021 due to the COVID-19 pandemic, and only outdoor public spaces were allowed to remain open. Thus, even under the heavy restrictions related to the pandemic there were still many outdoor-exercise opportunities that allowed the general public to remain active.

Recent research has focused on the impact of the COVID-19 pandemic and the resulting increasingly sedentary lifestyles among the population (Du et al., 2020; Dunton et al., 2020; Yamada et al., 2020; Ronkainen et al., 2021). There was still access to green space in Helsinki, and as Korpilo et al. (2021) found, in fact some forest areas nearby were over-crowded, which had a negative impact upon the environment. Informal sport is not often explicitly mentioned in policies: in Finland, for example, the Sports For All policy is still centered around organized sports focusing on diversity (Ministry of Education and Culture, 2018). It would therefore be useful to know where in urban spaces people are doing their PA. Urban spaces tend to be associated with a decreasing amount of green space and with discouraging people from exercising, which could fuel an increase in sedentary living (Triguero-Mas et al., 2022). Our focus here, however, is on how urban areas could still give access to a variety of outdoor sports.

A healthy city, as defined in the Zagreb Declaration (WHO Regional Office for Europe, 2009), is one that “provides conditions and opportunities that encourage, enable and support healthy lifestyles for people of all social groups and ages.” Li et al. (2022) conducted a review to distinguish any differences between green and non-green exercise: they found that green exercise did, in fact, have psychological advantages. Since then, social media has been used as a novel source of data to capture the usage of green space during the COVID-19 pandemic (Fagerholm et al., 2021; Korpilo et al., 2021; Cui et al., 2022). However, these studies omit other urban spaces in which informal PA takes place outdoors in fixed locations, such as outdoor gyms, skate parks, and table-tennis facilities. Moreover, outdoor informal sports such as cycling, kayaking, and walking do not take place in fixed locations. It would therefore be useful for municipalities to know where their populations were engaging in PA.

### 2.3. Previous usage of geotagged Twitter tweets

Geotagged Twitter messages have been successfully used in previous academic studies spanning many research topics, such as for analyzing political events (Tear, 2018) or human mobility (Jurdak et al., 2015). Huang and Carley (2019) refer to Twitter as a useful proxy for understanding people's mobility and social events. Tweets are naturally occurring data, the main purpose of which is not for analysis (Sloan and Morgan, 2015). This makes it potentially more suitable for further analysis given that data collected from online users as a by-product are said to be more reliable than survey or questionnaire data in which people might give false answers (McLaren and Shanbhogue, 2011). Tweets typically include a hashtag (#) alongside a phrase or term, which researchers follow spatially, temporally, and socially. Crampton et al. (2013), for example, followed “#LexingtonPoliceScanner” to find out how riots spread online in the US. They suggest that researchers go beyond the geotag and focus on the space associated with the geolocation and not just the point on the map, but it should be borne in mind that they were focusing on a localized event in one fixed location. We argue against this practice, as we have found that the geolocation, which for us is the most important aspect, is often omitted in secondary research analyses.

Key words that link “sports” and “Twitter” on web search engines, at least in the English language, typically identify research focusing more broadly on sports journalism, and on finding sports events. These results are linked to how the art of journalism has had to adapt to reporting news in different ways (Schultz and Sheffer, 2010), for example, or how social media can be used alongside humans as real-time sensors to find out when sporting events are taking place (Lanagan and Smeaton, 2011; Zhao et al., 2011b). Smith et al. (2019) point out that sports fans enhance their live sports viewing by using Twitter. This is a sentiment that has been noted in other studies, not only among fans but also among coaches, athletes, and sports media associates, originating in particular at the London Olympics in 2012, which was known as the “first social media Olympics” (Mann, 2012). In summary, the kind of volunteered geographic information mentioned above has long been used on other social-media platforms such as Flickr photos and Google Maps placemarks, which Crampton et al. (2013) call the “geographies of the geoweb.”

However, what is missing from these examples is the vital aspect of our research, namely where these tweets about sporting and news events are produced (Gritta et al., 2018), especially those in non-fixed locations. A similar result would be found in data from mobile network infrastructures, called transactional data, which Raun et al. (2023) cite has the potential to reveal activity spaces for second home usage, but still with a focus on the “where” and “when” of fixed locations. Although some sporting events are obvious, this is not the case with less widely known or news events, or informal leisure sports. The “where” of an event should be attached to a specific and unique location, and this spatial aspect (geolocation) is available as a geotag on Twitter (Middleton et al., 2018). We are aware that users post about and interact with sporting events, and we wonder if people in general also tweet about their own physical activities. Then “when” is not so important in our case, given that many previous studies have focused on this, such as Zhao et al. (2011a) as well as Lanagan and Smeaton (2011).

Previous geotagged Twitter tweet studies have begun to uncover land use functions including sport-related urban land use, such as Iranmanesh et al. (2022), but they do not mention which sporting activities and still omit the land cover type that the activity is occurring on. There should hence be more emphasis on the more specific “where,” which yields information for our study about which urban PA locations are popular in terms of user demand, and could help municipalities to keep up their maintenance or improve the usability of locations in the HMA. We are aware that the City of Helsinki already uses some people counters in specific outdoor PA spaces (Rämö, 2022), but although this example includes outdoor gym demand, it omits all other types of PA that might go on in the HMA. Members of the public have also noted that the maintenance of outdoor gyms could be improved, and that this could correlate directly with user demand. Therefore, Twitter messages would be a more well-rounded data source of skiing tweets among a wider population, as well as of other sports-related key words in the same locations.

## 3. Methods

### 3.1. Study area

**Figure 1:**
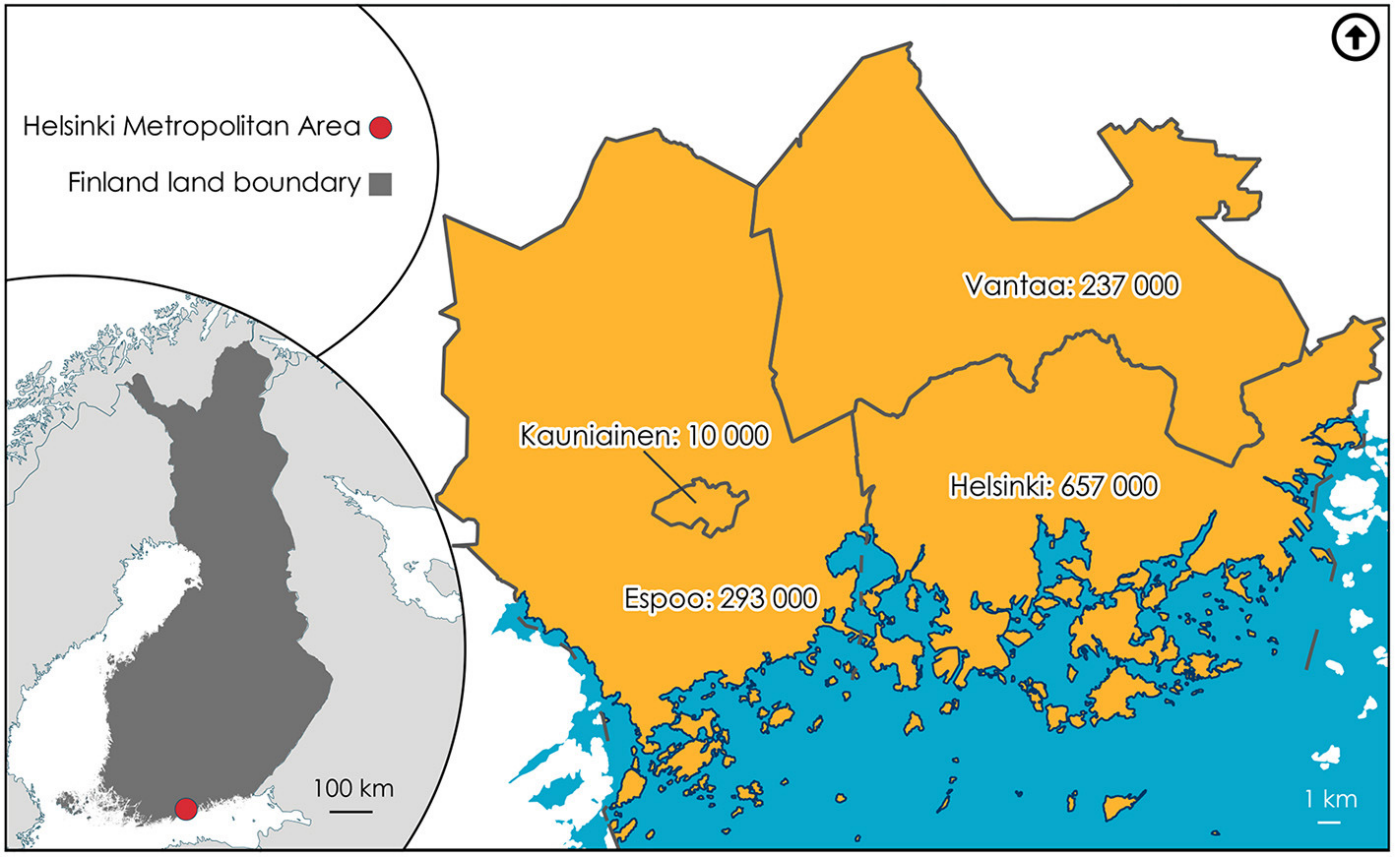
Helsinki Metropolitan Area: location and population counts.

We tested our approach in the case-study area, namely the Helsinki Metropolitan Area (HMA), with a total population of 1.2 million (Statistics Finland, 2020). Figure 1 shows each of the four areas as situated within the HMA. According to the LIPAS database, the HMA has a total of 5,084 sports facilities (LIPAS, 2022). Helsinki (2,775 facilities), Espoo (1,266), and Vantaa (1,002) are the top three municipalities in Finland in terms of sports facilities, the fourth being Kuopio (980) in the east, and the fifth Oulu (977) in the north (LIPAS, 2022). Kauniainen ranks 240th out of 308 municipalities, with 41 facilities in its 6 km^2^ area (LIPAS, 2022). We consider the HMA to be one borderless research unit, as residents typically live, work, and spend their leisure time freely across the four areas (Kepsu and Vaattovaara, 2008; Di Marino et al., 2018, 2021). The respective municipalities therefore need to know the locations in the HMA in which people engage in PA and spend their leisure time outdoors, as well as the respective demands. Finland's climate is of the continental type, being cold and with a long winter in the north and the interior, and relatively milder along the western and southern coasts. The HMA lies on the southern coast but still averages temperatures below freezing and has snow most winters, although the weather is becoming less predictable and these conditions cannot be guaranteed.

### 3.2. Data

We used two main types of data: (1) Twitter tweets data and (2) information from LIPAS sports facilities. The Twitter tweets comprise two combined secondary data sets: one was collected by the Digital Geography Lab at the University of Helsinki (DGL, 2022) and the other was part of an archive from the Digital OnLine Life and You (DOLLY) research project at the University of Kentucky (FloatingSheep, 2022). The total number of tweets amounted to 38,487,766 but it is worth pointing out that only a very small proportion (5.3%) of these contained geotags. The temporal range of both the data is between September 2006 and April 2020.

The LIPAS data on sports facilities are openly available for download and further analysis (LIPAS, 2022). LIPAS is a nationwide database containing information about sports facilities in Finland, which currently number around 42,000, and are all categorized. We chose five ski-related types of facility: (1) ski tracks (code 4402), (2) ski slopes and downhill ski slopes (code 4110), (3) ski-jumping hill for training (code 4310), (4) ski competition center (code 4630), and (5) cross-country ski parks (code 4640). We used these sports facilities locations in the results visualizations to find out if there were any matches between the sports and the tweet locations.

In the final visualization we display the individual tweets as points, a tactic that has been successful in other studies for activity mapping in terms of space (see Goličnik and Ward Thompson, 2010; Korpilo et al., 2021). It is an effective way of disseminating results to designers, for example, who are familiar with using visual material to influence decision-making. We additionally overlaid three land-cover, open polygon data sets from the Web Feature Service of the City of Helsinki: (1) lakes, (2) rivers, (3) sea, and (4) green space (Helsingin Karttapalvelu, 2022). The HMA boundary is as delineated in the Helsinki Region Map (Helsinki Region Environmental Services HSY, 2021).

### 3.3. Methodology

We followed the same methodology as Koivisto (2021) did, namely using both geotagged and geoparsed tweets, although we only used the geotagged tweets in this analysis. Geotagged tweets inherently contain location information, and are thus ready for further geographical analysis. Geoparsing is an additional method whereby a text location within the tweet is linked to geographical coordinates (Gritta et al., 2018). We used the programming language Python to identify the geotagged sports tweets, and the geographic information system software (QGIS) for manual data cleaning and analyzing the results visually.

Twitter stopped allowing followers to use the precise geotag feature in June 2019, although still keeping a general geotag feature, claiming that the majority of users did not use it (Hu and Wang, 2020). The precise geotag feature allowed the exact longitude and latitude coordinates to be utilized, whereas general geotags include a larger bounding box of a specific place such as a park or a city. We therefore include data from September 2006 to April 2020, although some tweets came after this date. Twitter still allows users to post a precise location through a third-party app such as Instagram or Sports Tracker, hence our results include these.

In order to extract the sporting information from the tweets we filtered the tweet texts for the sporting key words. To make the text in the tweets comparable, we then normalized the words by means of lemmatization, in other words returning to the dictionary form (Korenius et al., 2004). Next, we filtered the tweets by a list of sporting key words in both English and Finnish (see Table 2): tweets containing a word from this list were added to our data set. An additional data-cleaning task was to correct the filtered tweets manually to make sure that the action of skiing was being carried out. We only kept the tweets whereby the tweeter was currently engaged in or had just completed the activity (in our case: skiing). We removed the tweets if the tweeter was tweeting about another person, or if it was in the future tense, as we could not conclude with certainty that they had completed the action. We were then left with first-person action tweets. We started with 278 skiing tweets, but after the data cleaning we ended up with 150.

**Table 2:** Ski-related tweets: summarized statistics.

**Tweet language**	**Key word**	**Removed**	**Kept**
English	Ski, skiing	71	105
Finnish	*Hiihto, hiihtäminen, hiihtää*	57	45
	**Sum**	128 (46%)	**150** (54%)

We conducted a secondary analysis of tweets based on different key words that were located within a 100 m buffer zone as the remaining correct skiing tweets. We used the same 19 key words as in Koivisto's (2021) analysis, again restricting the languages to English and Finnish (their analysis included Estonian). We also removed the key words for ice-skating and ice-hockey in both the English and the Finnish tweets and kept only non-snow sports-related activities. Table 3 shows the resulting filtered sports key word options. We had 1,076 other sports-related tweets but following the data cleaning we removed 296 incorrect tweets, resulting in 745 correct ones. Tweets with more than one sports-related key word were counted again, hence the sum counts of key words is higher than the number of tweets. We counted the language of the key word and not the language of the tweet for the further analysis, for which we therefore had 798 sports-related key words.

**Table 3:** Non-snow-related sports tweets: summarized statistics.

**English key word**	**Removed**	**Kept**	**Finnish key word**	**Removed**	**Kept**
Basketball	5	2	*Koripallo, koris*	0	1
Bicycle, bike, biking, cycling	23	54	*Pyöräillä, pyörä, pyöräily, pyöräileminen*	10	23
Dance, dancing	40	33	*Tanssia, tanssi, tanssiminen*	10	4
Exercise, exercising, workout, training, sport, sporting	43	124	*Urheilla, treenata, treenaaminen, treeni, urheilu, liikunta*	21	23
Floorball	0	1	*Salibandy, sähly*	3	0
Football	29	9	*Jalkapallo, futis*	3	1
Gym	0	0	*Kuntosali*	2	4
Hike, hiking, trek, trekking	4	5	*Patikoida, patikointi, patikoiminen*	0	0
Run, running, jog, jogging	60	223	*Juosta, juoksu, juokseminen, lenkkeillä, lenkki, lenkkeily*	14	33
Sail, sailing, kayak, kayaking, canoe, canoeing, rowing	9	15	*Purjehtia, purjehdus, kajakki, meloa, melonta, soutaa, soutaminen*	0	0
Sweat, sweating	5	4	*Hiki, hikoilla*	2	4
Swim, swimming	10	11	*Uida, uinti, uiminen*	1	2
Tennis, badminton, squash, tabletennis	5	5	*Tennis, sulkapallo, squash, kössi, pingis, pöytätennis*	0	2
Volley, volleyball, beach volley	3	0	*Lentopallo, lentis*	0	0
Walk, walking	30	150	*Kävellä, kävely, käveleminen*	13	41
Yoga	0	21	*Jooga*	2	3
**English sum**	266 (28.8%)	**657** (71.2%)	**Finnish sum**	81 (36.5%)	**141** (63.5%)
			**Overall sum**	347 (30.3%)	**798** (69.7%)

## 4. Results and discussion

**Figure 2:**
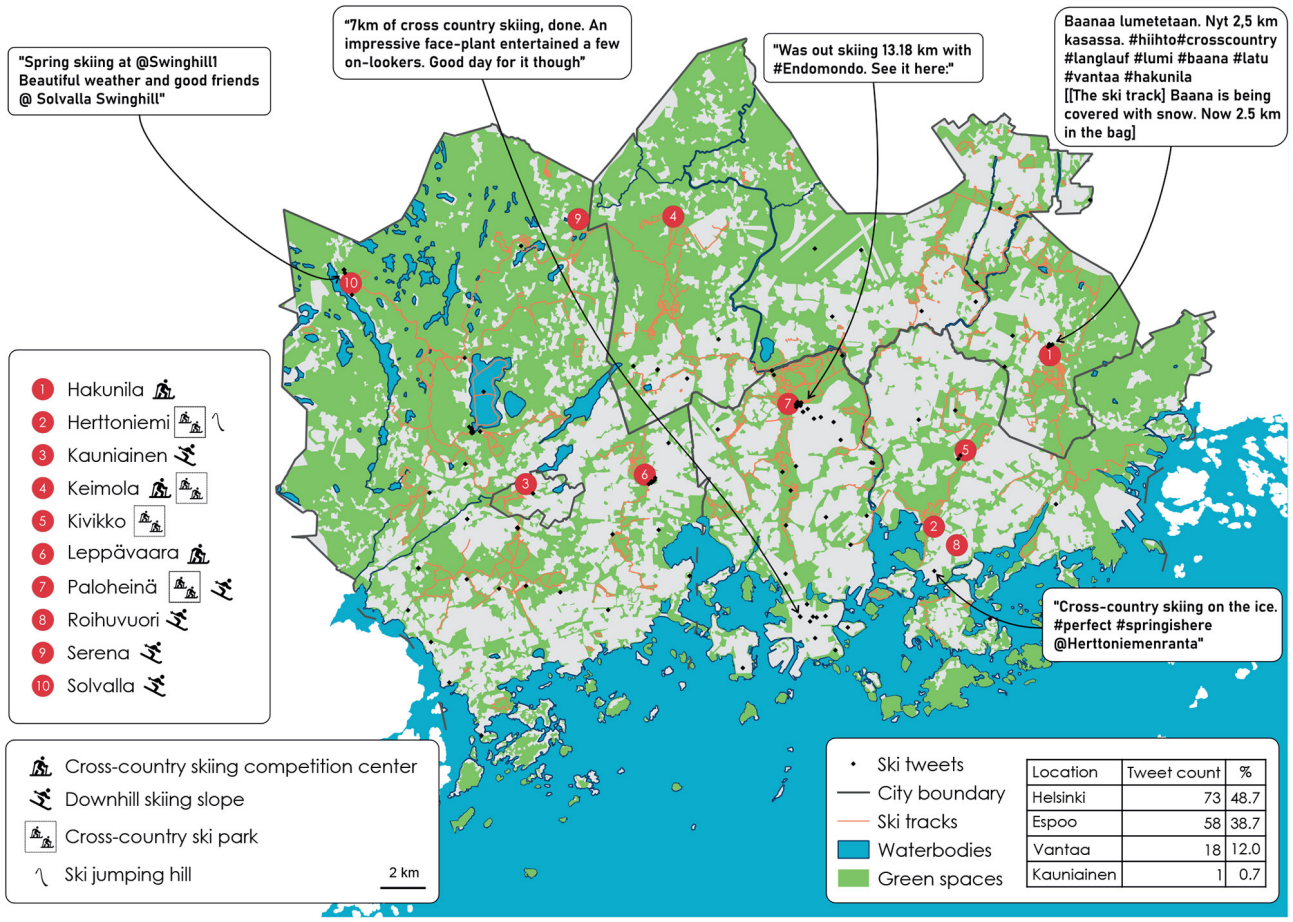
Skiing: tweet locations in the Helsinki Metropolitan Area.

**Figure 3:**
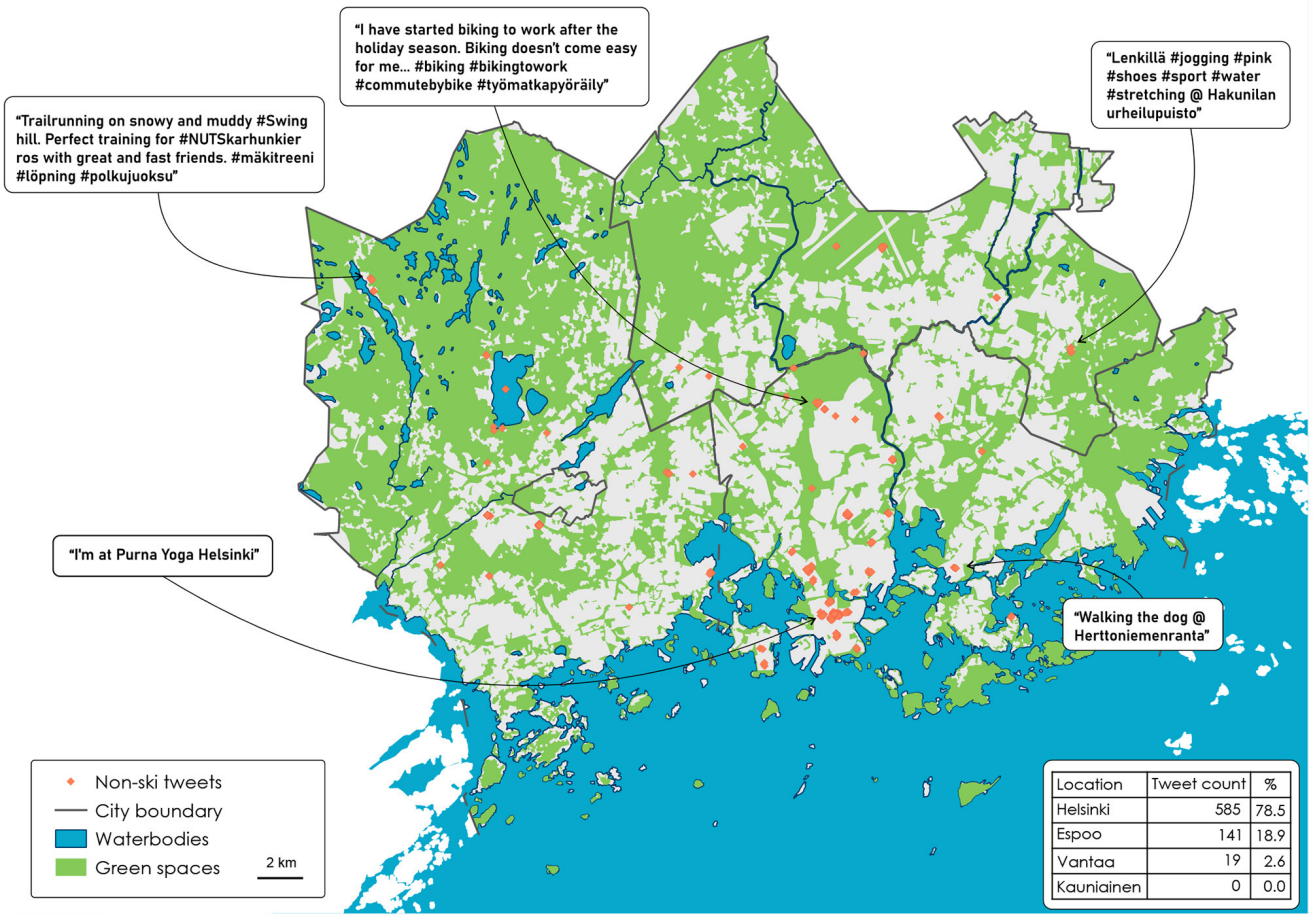
Other sports: tweet locations in the Helsinki Metropolitan Area.

Our main research purpose was to find out how geotagged tweets detect informal PA spaces in the Helsinki Metropolitan Area (HMA), complemented with the sub-purpose of identifying the type of urban public spaces in which people did their informal PA. In Section 2.2, we introduced green, blue, and street spaces, which we explore below. We categorize the results in terms of the need for municipalities to know where people are exercising and spending their leisure time outdoors, and the respective demand. First, we give the results for the ski tweets, and then we discuss the non-ski tweets in relation to them. We chose five locations with a tweet in both the ski and the non-ski locations to give an example of a tweet with different features for analysis, such as mentioning a location, not mentioning a location, or sharing a tweet *via* a third-party application. There is an emphasis on what other types of informal activities are going on in these spaces that diverge from the space's intended purpose. We further discuss how our findings could help municipalities, including sports planners and city planners, to improve the conditions in locations in which user demand is calculated through tweet popularity. Figure 2 shows the ski-related tweets, and Figure 3 shows the tweets in the same locations but also includes other sports-related key words. Finally, Table 4 shows the counts of the overlapping tweets with the blue, green, and street spaces.

**Table 4:** Land cover: summarized statistics.

**Land cover**	**Ski tweets counts**	**Non-ski tweets counts**
Green	76 (50.7%)	32 (4.3%)
Blue	5 (3.3%)	8 (1.1%)
Street	69 (46%)	705 (94.6%)
**Sum**	**150**	**745**

### 4.1. Ski tweets

#### 4.1.1. Ski locations

Figure 2 shows the results of the ski tweets. Spatial location is very important when analyzing tweets as the majority (62% of the 150 ski tweets) do not include a specific location within the text. However, it turns out that when they are added to a map output they are indeed in the correct location. This is a key finding because it is not self-evident from the text results. It is also a strong argument put forward by Sloan and Morgan (2015), who affirm that the geotag information is extremely valuable, in that the tweeter decides to tweet at some point during an event or at a location in real time. Although in Coakley's (2017) opinion informal sport participation is in decline, our study has shown that people do engage in informal cross-country skiing in the HMA, and they even tweet about it. As Figure 2 shows, there are overlaps with the ski tracks and the ski-related types of sports facility. There were tweets from close to six of the 10 ski-related facilities. With a capture rate of 60% resulting in 50 tweets ($\frac{1}{3}$ of total ski tweets), it is clear that these more formal facilities are also popular places for people to spend their free time. These results would not have come to light if only a textual analysis had been conducted—the “where” is thus a very important aspect of this analytical method. Additionally, two tweets mention the indoor ski tunnel at the Kivikko ski center in Helsinki. However, they are located in a street space even though the location of the ski center is within a green space. This shows that our analysis could be limited by the scale of the background data.

The fact that we only used pre-geotagged tweets raises a further discussion point in that we assume they are geotagged to a correct location that is mentioned within the tweet. However, some key definitions of informal sport in the current literature ignore the location, as shown in Table 1. Even though there is not yet a main emphasis on the location where sport happens, there were still two definitions that included it: Wheaton and O'Loughlin (2017) and Deelen et al. (2018) both use the term setting. Consequently, if we divide the HMA into green, blue, and street spaces, the tweet results, as shown in Table 4, are: 76 tweets (50.7%) in a green space, 69 (46%) in a street space, and five (3.3%) are in a blue space. This could be broken down further into tweets along ski tracks. Ski tracks in total have a combined length of 594.8 km, and they are maintained by the municipalities. Most of the paths are within a green space, accounting for 95.8% of all ski tracks. Hence, skiing in the HMA is also a form of green exercise in that the tracks go through parks and along forest paths. Additionally, people follow self-made tracks in the snow, which are not counted in the total length of tracks: they enable people to ski to the maintained tracks, or just close to their home since people can go cross-country skiing when there is enough snow on the ground.

Shanahan et al. (2016) make the overall claim that green spaces are important in terms of providing a location for physical activity, and in particular with an emphasis on green exercise with its better health and wellbeing benefits in comparison to PA in built-up or indoor environments. We do not focus on the specific health benefits in this article, but it is obvious that the HMA population has easy access to skiing tracks within green spaces. Next (Section 4.2.1), we consider which other sporting activities could be conducted in the same skiing locations when there is no snow.

#### 4.1.2. Ski demand

Finland offers opportunities to engage in outdoor exercise even in winter, skiing being one major hobby, to keep up with the recommended weekly target of 150 min of moderate-to-vigorous-intensity PA—which according to Bennie et al. (2017) 70% of adult Finns do not achieve. The tweet counts match the population of the municipalities, Helsinki having the most and also the highest population count (see Figure 1). Helsinki yielded 73 tweets, Espoo 58, Vantaa 18, and Kauniainen one. There are other ways of tweeting than writing directly within the Twitter site. Of the 150 ski tweets 60 came via Instagram (accounting for 40%), 39 (26%) via Endomondo (a social fitness network-tracking application that closed down at the end of 2020), five (3.3%) from Foursquare Swarm, which is a location diary shareable with friends, and two from Charity Miles (1.3%), a sports-tracking application for fundraising. This leaves 44 (29.3%) that were directly written in Twitter^1^. As Figure 2 shows, people living in the HMA do not need to leave their local area to engage in winter sports or to do PA outdoors. An example tweet is highlighted in Solvalla Swinghill Ski Center. The user has tagged (linked the ski center's Twitter username) the tweet so it could be said that the user was there at some point in time. From the perspective of this paper, then, the additional information of location within the text is also beneficial. An overall supply of cross-country skiing locations is thus beneficial in promoting healthy cities: if the municipalities know where the demand is in specific locations they could improve the maintenance of the tracks and facilities nearby, such as providing more garbage bins, benches to rest on, and toilet facilities, for example.

A negative aspect of improving facilities is that the routes could become overcrowded, and this has a negative impact upon the environment. Korpilo et al. (2021) found that this occurred during the use of nearby forests throughout the COVID-19 pandemic in the HMA. Another negative aspect that is often cited is that urban spaces have decreasing amounts of green space (Triguero-Mas et al., 2022). However, 57.4% of the HMA is green space measured according to the green space layer, and even then street spaces are still used for PA, as these tweet results show. It has also been found that engaging in PA improves overall health and wellbeing (Bowler et al., 2010), without having the extra stress of formal sports, such as having to be present at a regular time each week and the extra costs involved. Bell et al. (2015) also found that if the PA area is scenic and in natural surroundings, people are motivated to work on their overall wellbeing. In general, then, engaging in PA outdoors could be considered an inherently positive activity.

### 4.2. Non-ski tweets

#### 4.2.1. Non-ski locations

Next, we focused on what ski locations are used for when there is no snow. The green, blue, and street spaces are not just winter locations as they are open public spaces, which is very common in Finland. Figure 3 shows the results for the non-ski tweets. Following the green, blue, and street analysis, as shown in Table 4, 32 tweets (4.3%) were in a green space, eight (1.1%) tweets were in a blue space, and 705 (94.6%) in a street space. This is a big contrast to the ski-tweet results. The example tweet in the Espoo figure is for the Swinghill downhill ski center, and the tweeter is tweeting about trail running. Multi-use outdoor PA spaces are not unique in Finland, but they are plentiful in comparison to other countries, which was particularly evident during the peak of the COVID-19 pandemic: some countries, such as France, even restricted the use of urban parks (Geneletti et al., 2022). In England, aside from the pandemic, the Countryside and Rights of Way Act 2000 gives the legal right of public access only to about 8% of the countryside, which severely limits opportunities for informal PA (Horton, 2022). Finnish forest areas, such as Central Park (“Keskuspuisto” in Finnish) in Helsinki, have specific paths to be used for skiing during snowy weather (when cycling is not allowed), and during non-snowy weather there are specific paths for cycling, horse riding and walking. However, our results show only one tweet in this location. Of the 745 tweets, 362 (48.6%) mention a location. It is clear from this result that if the geotag were missing, the location of the remaining 51.4% of the tweets would remain a mystery, even with the additional step of geoparsing because there is no location to geoparse. This also reflects Hu and Wang's (2020) concern about the removal of Twitter's precise geotagged tweets and its research consequences.

Within the tweet location the results for non-ski tweets reveal a cluster in Helsinki city center, which is the largest in the southern part of the map. These tweets have the same coordinates (60.1708, 24.9375), which is the center of Helsinki, and therefore are located here when a tweeter tags Helsinki as a more general location than where they are located. This cluster comprises 220 tweets, with another 123 surrounding it (in total 46%). This type of tagging behavior is not so useful for further analysis because it means losing some of the data, but it benefits the tweeter in not revealing the exact location. Mapping individual points has proven to be beneficial for the mapping of open spaces by researchers such as Goličnik and Ward Thompson (2010), who mapped larger public parks and how their design limited user behaviors. They concluded that larger open spaces were used for team sports such as football and that paths were mainly used for walking or cycling, thus implying that urban design dictates how people should behave in a space. An example of unusual behavior in a park is then to cycle across a grass field if there are paths around the edge. This behavior setting extends to the inappropriate use of green spaces: if many people similarly misbehave, the result would be an area with muddy informal paths where the grass has been removed, which in turn would lead to environmental degradation. This scenario extends to urban forests: Korpilo et al. (2021) reported the overuse of nearby forests during the COVID-19 period in Helsinki.

#### 4.2.2. Non-ski demand

There were 745 non-ski tweets: 585 in Helsinki, 141 in Espoo, 19 in Vantaa, and none in Kauniainen. In comparison to the ski results, there was a higher sum of tweets in Helsinki compared to the other three municipalities, accounting for 78.5% of all the non-ski tweets. In terms of where the tweets are posted from, 440 (accounting for 59.1%) were via Instagram, 103 (13.8%) via Endomondo, 27 (3.6%) via Foursquare Swarm, and 77 (10.3%) via Charity Miles. The remaining 98 (13.2%) were posted directly from Twitter. Figure 3 shows five examples of tweets representing four different types of sport, but in the same location as the examples of ski tweets given in Figure 2 (trail running, biking, jogging, walking, and yoga). Environmental decisions could promote healthy living in specific urban-design and social settings (Ward Thompson, 2013). If it is known where people engage in PA, municipalities could plan more effectively where to put more facilities, such as bike racks and starting routes from public-transport stops. Another example is where people follow specific routes created by the municipalities to both control and increase outdoor PA: decisions have to be made such as where to place garbage bins, toilets, and signposts to specific routes. There has recently been increasing interest in designing walkable cities as a consequence of the COVID-19 pandemic (Fagerholm et al., 2021). Knowing the tweet demand may help municipalities to improve locations to make people feel more comfortable and willing to spend longer outside.

The results in combination create a new and more comprehensive data set concerning the demand for informal sporting locations that the municipalities in the HMA could utilize to better allocate funding for improvements, such as those facilities mentioned above. In relation to Jeanes et al. (2019) who focus on sports policies, the data and information that have been produced from the tweet locations can serve as a tool that helps municipalities to include the practices of knowledge-based management into their daily work. The information can be used by city or sports planners when they know where informal sports and PA spaces are being utilized in order to help plan facilities and cities in such a way that access is equal for the population. This is a key idea and result as we have shown that a wide variety of sports are conducted in the same outdoor locations, even when restricting our study to 16 sports keywords. This further means that the population already feel welcome to use these locations at any time or location which formal sports might restrict, but improvements can still be made so that a wider population has access to outdoor PA spaces.

## 5. Conclusion

Gilchrist and Wheaton (2011) note the difficulty in finding out participation rates for informal sports, and we took this as an opportunity to gather a user-generated data set of Twitter tweets. We have shown that, even with a very small subset of an already limited portion of data that is geotagged, it is possible to obtain sound results for further analysis. We detected informal spaces for skiing activity that might otherwise be ignored in efforts to understand where people engage in physical activities in the HMA. Improving the health of the population through facilitating engagement in informal sports is a simple and cost-effective form of intervention to reverse the increase in illnesses related to physical inactivity, which costs the country billions annually. We further focused on other types of informal activities going on in these spaces that differ from the intended purpose, to shed light on the PA that goes on in the same locations when there is no snow.

The HMA is a very inclusive area from a sports facility perspective and therefore allows the population to have many different opportunities for maintaining active and healthy lifestyles. Our results support the suggestion put forward by Neuvonen et al. (2018) that the HMA has been designed and maintained so that everyone has equal access to outdoor spaces regardless of whether or not they realize they like to be outdoors doing PA. Informal sports do not require regular monetary or time commitments, and are therefore suitable for a larger proportion of the country's population. Informal sports often require less regular monetary or time commitment than organized sports and therefore may be suitable for a larger proportion of the country's population. Our research can therefore be expanded to a national level and a wider variety of sports to capture any variations between municipalities to locate which populations are less active in using social media to post about their PA. A further research aspect could be the impact of climate change upon the skiing possibilities in the HMA. Our results have already shown that the population do conduct other types of PA in the same locations, but it would be interesting to find the types of PA during a less snowy winter.

Finally, future research should focus on detecting PA spaces using the 90-plus percent of tweets that do not have a geotag. This involves complex geoparsing to add the missing locational information (Gritta et al., 2018), and in potentially revealing different demands of informal spaces that are not currently known could be a valuable method to test. It is difficult to create national policies focusing on informal sports because it means taking a step back and letting the population do their own thing away from formal sports facilities. The more traditional data-collection methods involve surveys, which are open to exaggeration on the part of the participants, or the giving of false information, as McLaren and Shanbhogue (2011) found. It is therefore more difficult to know how many people, and more specifically, who (such as the age, gender, or ethnicity) use the informal outdoor facilities, and if they even bring about change in sedentary lifestyles. Overall, we conclude that Twitter may be a beneficial tool in detecting year-round spaces for informal physical activity. The benefits could extend to city planners and sports planners aiming to improve informal sports facilities based on user demand, and thereby promote the development of healthy cities.

## Data availability statement

The original contributions presented in the study are included in the article/supplementary material, further inquiries can be directed to the corresponding author/s.

## Author contributions

CL, EE, SK, and PM: study design, reviewed the results, and approved the final version of the manuscript. CL: draft manuscript preparation. EE and SK: methodology, preparation of the results, and the analysis. PM: acquiring funding, supervision, and project coordination. All authors contributed to the article and approved the submitted version.

## Footnotes

^1^The results sum to 100 when accounting for 0.3 recurring in the percentages.
